# Defensive symbiosis against giant viruses in amoebae

**DOI:** 10.1073/pnas.2205856119

**Published:** 2022-08-29

**Authors:** Patrick Arthofer, Vincent Delafont, Anouk Willemsen, Florian Panhölzl, Matthias Horn

**Affiliations:** ^a^Centre for Microbiology and Environmental Systems Science, University of Vienna, 1030 Vienna, Austria;; ^b^Doctoral School in Microbiology and Environmental Science, University of Vienna, 1030 Vienna, Austria;; ^c^Ecologie et Biologie des Interactions Laboratory, UMR CNRS, Université de Poitiers, 7267 Poitiers, France

**Keywords:** giant virus, chlamydia, protist, amoeba, protective symbiosis

## Abstract

Protists are important regulators of microbial communities and key components in food webs with impact on nutrient cycling and ecosystem functioning. In turn, their activity is shaped by diverse intracellular parasites, including bacterial symbionts and viruses. Yet, bacteria–virus interactions within protists are poorly understood. Here, we studied the role of bacterial symbionts of free-living amoebae in the establishment of infections with nucleocytoplasmic large DNA viruses (Nucleocytoviricota). To investigate these interactions in a system that would also be relevant in nature, we first isolated and characterized a giant virus (Viennavirus, family Marseilleviridae) and a sympatric potential *Acanthamoeba* host infected with bacterial symbionts. Subsequently, coinfection experiments were carried out, using the fresh environmental isolates as well as additional amoeba laboratory strains. Employing fluorescence in situ hybridization and qPCR, we show that the bacterial symbiont, identified as *Parachlamydia acanthamoebae*, represses the replication of the sympatric Viennavirus in both recent environmental isolates as well as *Acanthamoeba* laboratory strains. In the presence of the symbiont, virions are still taken up, but viral factory maturation is inhibited, leading to survival of the amoeba host. The symbiont also suppressed the replication of the more complex *Acanthamoeba polyphaga mimivirus* and *Tupanvirus deep ocean* (Mimiviridae). Our work provides an example of an intracellular bacterial symbiont protecting a protist host against virus infections. The impact of virus–symbiont interactions on microbial population dynamics and eventually ecosystem processes requires further attention.

Protists are ubiquitous microbial eukaryotes found in virtually any environment ranging from natural aquatic or terrestrial ecosystems to engineered environments (1). As primary producers and predators, protists are important components of trophic networks. They facilitate the transfer of organic matter and energy from lower microbes to animals, and they represent key regulators of bacterial community composition. Heterotrophic and phototrophic protists alike are intimately associated with a diversity of bacteria and viruses.

Viruses shape protist populations, with major impact on entire ecosystems as in the case of algal blooms and demise (2). A widespread yet enigmatic group of viruses infecting protists are the giant DNA viruses (nucleocytoplasmic large DNA viruses; Nucleocytoviricota) (3–7). In terms of particle and genome size, these viruses are comparable to prokaryotes and small eukaryotes. The large degree of mosaicism in their genomes suggests an intimate association with both eukaryotes and prokaryotes throughout their evolutionary history (8).

In contrast to the lytic giant viruses, bacterial symbionts of protists can either be mutualists, parasites, or commensals. While protists are frequently associated with diverse bacterial endosymbionts, functions and potential benefits of these intracellular bacteria are largely unknown (9–11). Importantly, the interaction between endosymbionts and viruses infecting protists are hardly studied and poorly understood (12). What are the consequences of such coinfections for the partners involved, and by extension for the ecological role of protists? To study this in an experimental system that would also be relevant in nature, we set out to freshly isolate and characterize a giant virus and a cooccurring amoeba host infected with bacterial endosymbionts.

## Results and Discussion

### A Sympatric Giant Virus, Amoeba Host, and Bacterial Symbiont.

**Fig. 1.**
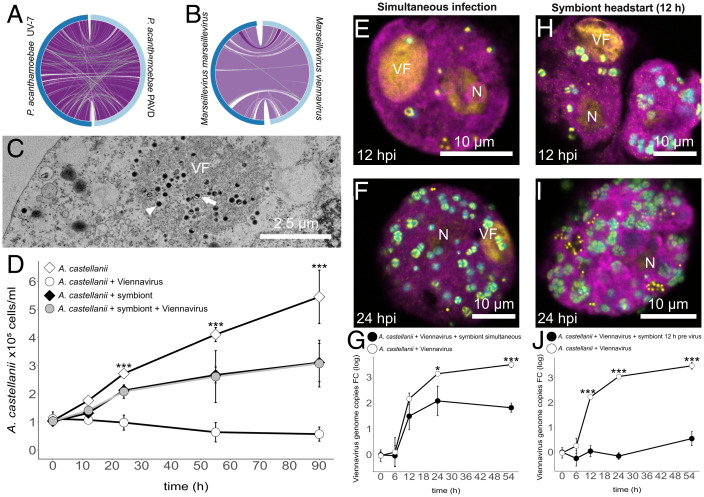
Coisolation of Viennavirus, *P. acanthamoebae*, and *A. hatchettii* and evidence for symbiont-mediated virus inhibition. (*A* and *B*) Near colinearity of the genomes of Viennavirus with *Marseillevirus marseillevirus*, and of *the Parachlamydia* symbiont PAVD with *P. acanthamoebae* UV-7. (*C*) Transmission electron micrograph; cytoplasmic viral factory (VF) of Viennavirus in *A. castellanii*, containing filled (arrow head) and half-filled (arrow) viral particles. (*D*) Influence of the *Parachlamydia* symbiont on amoeba host fitness during Viennavirus infection. Note that cell counts included infected, uninfected, and dying cells. As dead cells often remain intact for extended time periods (6), the host cell number never reached 0. Statistical tests were carried out with a two-sided ANOVA (***P < 0.001). *A. castellanii* simultaneously infected with Viennavirus and the *Parachlamydia* symbiont: FISH images (*E*) 12 hpi and (*F*) 24 hpi and quantification of viral particles with qPCR (*G*). *A. castellanii* infected with the *Parachlamydia* symbiont 12 h before the addition of Viennavirus: FISH images (*H*) 12 hpi and (*I*) 24 hpi and quantification of viral particles with qPCR (*J*). In all FISH images amoeba cells appear in magenta, nucleus (N) and viral factories (VF) in yellow, and bacteria in cyan. Statistical analysis for qPCR was carried out with two-tailed unpaired Student’s *t* test (*P < 0.05 and ***P < 0.001).

Using *Acanthamoeba castellanii* as a surrogate host, a lytic virus was isolated from activated sludge of a wastewater treatment plant previously shown to contain diverse giant viruses (3–5, 7, 13). Genome sequencing and transmission electron microscopy identified this virus as a new member of the giant virus family Marseilleviridae (4, 14), tentatively named *Marseillevirus viennavirus* (Viennavirus) (Fig. 1 *B* and *C*; methods are provided in SI Appendix). An *Acanthamoeba hatchettii* isolate was obtained from the same sample. The amoeba contained bacterial symbionts, identified by genome sequencing as a novel *Parachlamydia acanthamoeba* strain (PAVD; Fig. 1*A*). These ubiquitous bacteria, also referred to as environmental chlamydiae, are well-known for their evolutionary ancient and obligate intracellular lifestyle in diverse hosts (15).

### Chlamydial Symbionts Inhibit Viennavirus Replication.

**Fig. 2.**
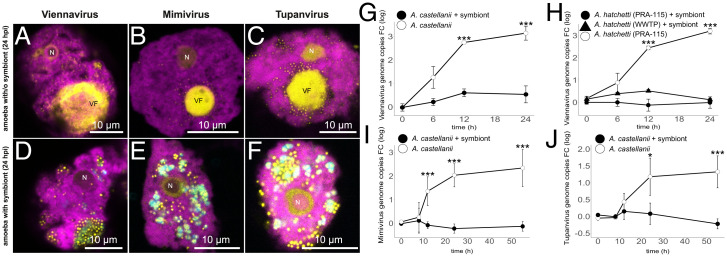
*P. acanthamoebae* represses Viennavirus, Mimivirus, and Tupanvirus replication. Fluorescence micrographs showing (*A*) Viennavirus, (*B*) *Acanthamoeba polyphaga mimivirus*, and (*C*) *Tupanvirus deep ocean* infection of *A. castellanii* in the absence of the symbiont. Amoeba carrying *Parachlamydia* symbionts and infected with (*D*) Viennavirus, (*E*) Mimivirus, and (*F*) Tupanvirus. In all FISH images the amoeba cells appear in magenta, nucleus (N) and viral factories (VF) in yellow, and bacteria in cyan. The progression of infection was monitored by qPCR for (*G*) Viennavirus in *A*. *castellanii*, (*H*) Viennavirus in the sympatric *A. hatchetti* isolate, and in the laboratory strain *A. hatchetti* PRA-115, (*I*) Mimivirus in *A*. *castellanii*, and (*J*) Tupanvirus in *A*. *castellanii*. Statistical analysis was carried out with two-tailed unpaired Student’s *t* test (*P < 0.05 and ***P < 0.001).

Like other Marseilleviridae (14), Viennavirus infection in *A. castellanii* is highly lytic, causing rounding of cells and amoeba lysis within 12 h postinfection (hpi) and leading to demise of the host population within 55 h (Fig. 1*D*). Surprisingly, when we infected the sympatric *A. hatchetti* isolate, naturally containing the parachlamydial symbiont, no viral factories were observed. Consistent with this, no viral replication measured by qPCR was detected (Fig. 2*H*). To understand whether the symbionts were the cause of Viennavirus inhibition, we tried to produce aposymbiotic cultures of *A. hatchetti*, albeit without success. As an alternative approach, purified symbionts were transferred to *A. castellanii* and another *A. hatchetti* strain (PRA-115), which led to the establishment of the symbionts in these amoeba cultures. When subsequently infected with Viennavirus, no viral factories were observed in both *Acanthamoeba* species that now contained the symbiont. Viennavirus replication was significantly repressed, mirroring our findings for the naturally cooccurring host amoeba. In contrast, symbiont-free controls showed viral factory formation and viral replication (Fig. 2*A*).

To analyze the influence of Viennavirus, the symbiont, and the interplay between both on their amoeba host, amoeba growth was monitored during infection experiments. The increased doubling time of *A. castellanii* carrying the symbionts compared to symbiont-free amoeba (22.7 h vs. 17.3 h) (Fig. 1*D*) indicates that the *Parachlamydia* symbiont has a negative impact on host fitness. This is consistent with the parasitic lifestyle of most chlamydiae, all of which are able to tap the host’s ATP pool and are auxotrophic for diverse metabolites (16). When infected solely by Viennavirus, amoeba cell numbers decreased (Fig. 1*D*). Notably, amoeba stably associated with the symbiont showed the same growth rate in the presence and absence of Viennavirus, maintaining cell division despite viral challenge (Fig. 1*D*). Overall, the symbiont positively affected the fitness of the amoeba host in the face of the lytic Viennavirus and can thus be considered a mutualist during viral predation.

### Interference with Viral Factory Maturation and Virion Production.

We infected amoebae simultaneously with the giant virus and the symbiont. This experimental setup led to the formation of viral factories observed at 12 and 24 hpi, notably along with the presence of intracellular symbionts (Fig. 1 *E* and *F*). The replication of Viennavirus was diminished by approximately one order of magnitude compared to the symbiont-devoid control but not blocked completely (Fig. 1*G*). Viennavirus is thus able to enter amoeba cells in the presence of the symbiont, yet virus replication is impaired (17).

The symbiont was next added to naïve amoebae 12 h prior to virus infection, a time point which coincides with major changes in the host cell and the establishment of the inclusion, the phagosome-derived vacuole containing the bacteria (18). Viral factories were seen 12 but not 24 h after viral infection (Fig. 1 *H* and *I*). Contrary to the simultaneous infection setup (Fig. 1*G*), no Viennavirus replication was observed over the time course of the head-start experiment (Fig. 1*J*). Thus, in this situation, early stages of viral factories can be formed, but their maturation and subsequent virion assembly are inhibited.

In summary, these findings, together with the notion that Marseilleviruses exploit different entry paths (17), indicate that viral replication but not viral entry is blocked, even when the amoeba host has been stably associated with the symbiont (Fig. 2 *G* and *H*). After viral entry, direct contact between the virion or the viral factories with the chlamydial symbionts seems unlikely as the bacteria are confined to host-derived inclusions (19). It is thus most parsimonious that host cellular pathways are manipulated through bacterial effectors (18). The extent of this effect is dependent on the relative timing of symbiont and virus infections. In the most natural situation, in which the symbiont is present before viral attack, this leads to a cellular environment effectively preventing viable viral factory formation and as a consequence virion production.

### *P. acanthamoebae* Represses Replication of Different Giant Viruses.

We infected *A. castellanii* carrying the *Parachlamydia* symbiont, with *Acanthamoeba polyphaga mimivirus* and *Tupanvirus deep ocean*. While Viennavirus belongs to the Marseilleviridae, Mimivirus and Tupanvirus are Mimiviridae. Mimivirus was the first described giant virus and serves as a “model system” (3). Tupanvirus is one of the most complex known viruses, and it was suggested that its replication is more independent from the host cell (7). While viral factories were observed for both viruses in the absence of the symbiont (Fig. 2 *B* and *C*), no such structures were seen after Mimivirus or Tupanvirus infection when the symbiont was present (Fig. 2 *E* and *F*). Consistent with the lack of viral factories, no Tupanvirus or Mimivirus replication measured by qPCR was detected in the presence of the symbiont (Fig. 2 *I* and *J*). The *Parachlamydia* symbiont is thus able to protect its amoeba host from evolutionary and structurally distinct giant viruses.

In this study, we documented an example of symbiont-mediated defense against viral infection in unicellular eukaryotes. The protective effect conferred by the intracellular symbiont *P. acanthamoebae* was observed with three distinct giant viruses, which differ considerably with respect to infection cycle and replication strategy (20). With amoeba frequently carrying bacterial symbionts (11, 19), symbiont-mediated protection against viruses might be a broader phenomenon with important consequences for all players involved.

The symbionts gain a selective advantage by improving host fitness in the face of viral predation, compensating for the burden caused by their auxotrophies and requirements for host metabolites. For giant viruses, competition with intracellular bacteria causes the need to adapt not only to interact with the amoeba host and virophages but also with bacteria present in the same intracellular niche (6, 8). Amoeba have been considered as “evolutionary melting pots” facilitating gene transfer between bacteria and viruses (4). The necessity to overcome symbiont-mediated inhibition might promote gene acquisition by giant virus genomes, providing an evolutionary pressure consistent with their large genome sizes and the accordion-like model of giant virus evolution (8).

For protists, the association with bacterial symbionts increases their chance for survival in the face of viral predation. In the absence of any effective antiviral defense system, protists are lysed, releasing organic carbon available for other microbes. In the presence of protective symbionts, however, protist survival would favor the transfer of organic matter from microbes to multicellular organisms. Taken together, symbiont-mediated giant virus inhibition can alter protist population dynamics and affect the ecological role of protists and, by extension, nutrient flow in entire ecosystems.

## Materials and Methods

Infection experiments, qPCR, and fluorescence in situ hybridization (FISH) were done as described (6, 10). Detailed descriptions are provided in *SI Appendix*.

## Supplementary Material

Supplementary File

## Data Availability

Sequence data have been deposited in GenBank/ENA/DDBJ (Bioproject PRJNA799241) (21).
