# Supplementary material for: Defensive symbiosis against giant viruses in amoebae
Source: Proc Natl Acad Sci U S A. 2022 Aug 29;119(36):e2205856119. doi: 10.1073/pnas.2205856119 (PMC9457554; doi:10.1073/pnas.2205856119)
Supplement: Supplementary File [file pnas.2205856119.sapp.pdf]

Defensive symbiosis against giant viruses in amoebae

Patrick Arthofer, Vincent Delafont, Anouk Willemsen, Florian Panhölzl, Matthias Horn

Supporting information

Extended Material and Methods

Isolation and cultivation of microorganisms

Two liters of activated sludge were sampled at the surface of a nitrifying reactor in March 2017 at the wastewater treatment plant in Klosterneuburg (Austria) (48°17'38.8"N 16°20'30.3"E) and transferred into sterile borosilicate bottles. The activated sludge was brought to the laboratory for immediate processing.

Amoebae were isolated by pouring an aliquot (~1 ml) onto a non-nutrient agar plate (NNA PAS: 1 g/l sodium citrate, 0.4 mM $CaCl_2$, 4 mM $MgSO_4$, 2.5 mM Na_2HPO_4, 2.5 mM KH_2PO_4,15 g/l agar) covered with *E. coli* strain JW5503-1 Δ*tolC732::kan* suspension (1). Once amoeba growth was observed, they were subcultured by transferring a piece of agar onto a new NNA PAS plate seeded with *E. coli*. Subsequently, the symbiont-containing *Acanthamoeba hatchetti* isolate was cultured in monoxenic liquid cultures, prepared with liquid PAS buffer containing *E. coli* (~5 x 10^8 cells/ml).

Acanthamoeba castellanii Neff (ATCC 50373), *Acanthamoeba lenticulata* (ATCC 50705), *Acanthamoeba byersi* (PRA-411) were grown axenically on Peptone-Yeast Extract-Glucose (PYG: 1 g/l sodium citrate, 0.4 mM $CaCl_2$, 4 mM $MgSO_4$, 2.5 mM Na_2HPO_4, 2.5 mM KH_2PO_4, 20 g/l proteose peptone, 1 g/l yeast extract, 0.1 M glucose) in 25 cm^2 flasks at 20 °C. *Acanthamoeba hatchetti* (PRA-115) was grown axenically on TSY medium (TSY: 30 g/l trypticase soy broth, 10 g/l yeast extract) in 25 cm^2 flasks at 20 °C. *Vermamoeba vermiformis* (ATCC 50237) was grown axenically on SCGYEM (11 mM NaH_2PO_4, 5.9 mM KH_2PO_4, 2.5 mM NaOH, 10 g/l casein, 5 g/l yeast extract, 13.87 mM glucose, 5 % of final volume: fetal calf serum). *Naegleria clarki* (CCAP 1518/14) was grown monoxenically on *E. coli* in PAS buffer.

E. coli strain JW5503-1 Δ*tolC732::kan* was cultured in liquid LB-medium (10 g/l NaCl, 10 g/l tryptone, 5 g/l yeast extract) overnight at 37 °C, 200 rpm and stored at 4°C.

Viral screening and isolation

Activated sludge samples were distributed into 5 ml aliquots, treated overnight at 4 °C under gentle agitation, with ampicillin (100 µg/mL), amikacin (100 µg/mL), rifampicin (200 µg/mL) and amphotericin B (25 µg/mL). Samples were subsequently homogenized by ten passages through a 26 gauge needle and through a 1.2 µm filtration step.

To screen for viruses, 10^5 amoeba trophozoites were distributed in each well of a 24-well plate, in PYG medium supplemented with ampicillin (50 µg/mL) and amikacin (25 µg/mL). Wells were inoculated with either 10 or 100 µL of pre-treated sludge and incubated at 20°C. After 7 days, 100 µL from the infected wells were transferred to wells with fresh amoeba of the corresponding type. Amoeba cultures were screened for cytopathic effects for several passages.

To isolate viral particles, amoeba were collected by scraping, and centrifuged for 15 min at 10,000 g. The supernatants were discarded, and the pellets were resuspended in 1 ml PAS buffer. To release viral particles from the remaining trophozoites, a mechanical lysis step was implemented by vortexing of the cell suspension at full speed for 2 min in Lysing Matrix E tubes (FastPrep®). To purify viruses from cell debris, samples were filtered through a 0.8 µm filter. Subsequently, the giant virus particles were preserved at -80 °C in PAS buffer.

In order to obtain clonal virus populations, virus particles were isolated by dilution-to-extinction by transferring single infected *A. castellanii* cells to uninfected amoeba cultures. After confirmation of viral propagation, one amoeba cell culture was expanded and viruses were isolated as described above.

Co-infection experiments

The lab strains *Acanthamoeba hatchetti* PRA-115 and *Acanthamoeba castellanii* were infected with the *Parachlamydia acanthamoebae* PAVD symbiont originating from the *Acanthamoeba hatchetti* isolate recovered in this study. All amoeba could be maintained in continuous culture together with the symbiont. Co-infection experiments were carried out in 24-well-plates at 20 °C. Viral particles were added at a multiplicity of infection (MOI) of 1 (based on quantitative PCR and ddPCR estimates, see below), and the infection was synchronized with a 30 min centrifugation step at 1,000 g. To avoid re-infections, the medium was replaced after the centrifugation. Symbiont-free cell cultures were used as positive controls, while cell cultures with the symbiont only served as negative controls. At each sampling time point, cells were detached and suspensions were centrifuged for 15 min at 10,000 g. Subsequently, the supernatant was discarded, and the pellet was stored at -20 °C for subsequent DNA extraction.

In a separate experiment, amoeba cultures were freshly infected with the symbiont 12 hours prior to the viral infection. For this, an MOI of 30 was used to ensure that every single amoebae cell contained at least 1-5 symbionts at 12 hpi.

Co-infection experiments with amoeba simultaneously infected with the *Viennavirus* and the *Parachlamydia* symbiont, were carried out as described above with the only difference that the symbiont (MOI 30) was added together with the giant virus (MOI 1) to naive amoeba cell cultures.

The amoeba fitness during a *Viennavirus* infection was evaluated via counting of attached trophozoites using hemocytometer (Fast ready 102) at 0, 12, 24, 55 and 90 hours post experiment start. Counting was carried out using three aliquots per amoeba culture.

All co-infection experiments were carried out in at least duplicates and repeated three times.

Fluorescence in situ hybridization (FISH)

Fluorescence in situ hybridization (FISH) was performed with amoebae trophozoites, which were collected and transferred to a well of a teflon-coated 10-well slide. Cells were left to adhere for 45 min and then fixed using 4 % paraformaldehyde for 10 min at room temperature. After rinsing, samples were dehydrated in ethanol baths (50 %, 75 %, 96 % ethanol for 3 min each). To each of the slides 10 µl of the hybridization buffer (90 mM NaCl, 20 mM Tris-HCl, 0.01 % SDS, 25 % formamide) and 1 µl of the probes Euk516 (labeled with the fluorophore Cy5) (2), a mixture of $Eub338_{1-3}$(Fluos) (3, 4), and 4,6-diamidin-2-phenylindole (DAPI) (1 µg/ml) were added. For the *Naegleria* culture the custom-designed probe Nag1088 (Cy5) was used instead of the Euk516 probe. Additionally, the *Parachlamydia* specific probe Para581 (Cy3) was used to visualize the symbionts. Hybridizations were performed in moist chambers at 46 °C for 1.5 hours. Subsequently, slides were washed with 12 µl of the washing buffer (1 mM Tris-HCl, 0.25 mM EDTA and 0.149 M NaCl) and kept in the washing buffer for 10 min at 46 °C. Slides were ultimately washed for 10 s in cold water (~4 °C) and air-dried. The slides were observed using a confocal laser-scanning microscope (Leica SP8).

Transmission electron microscopy

Amoeba were centrifuged for 10 min at 3000 g. The pellets were fixed in 2.5 % glutaraldehyde (Agar Scientific) in 0.05 M phosphate buffer (Na_3PO_4) (Merck) for 1 hour at room temperature and washed 3 times for 10 min with the same buffer. The samples were fixed in 2 % osmium tetroxide (EMS/Science Services) in aqueous solution for 1 hour and washed 3 times for 10 min in ultrapure water (ddH_2O). Dehydration was done with an ascending serial dilution of ethanol (30, 50, 70, 90 and 100 %, 15 min each) and 3 times 100 % acetone (5, 7 and 10 min). The dehydrated samples were then covered with a mixture of acetone and EPON resin (Agar 100, Agar Scientific); two times each with a mixing ratio of acetone:EPON of 3:1 (10 min and 1 hour) and 1:1 (20 min and overnight). All steps were performed on a shaker at room temperature and if necessary followed by

a 3 min centrifugation at 3000 g (prior to exchange of liquids). Finally, the samples were covered twice in pure EPON resin for 1 hour (the second time left in a desiccator at 250 mbar), transferred in embedding tubes, covered with fresh EPON resin and dried for 1 hour in a heating cabinet at 45 °C and then at 60 °C for 3 days. Ultra-thin sections (produced with a Leica EM UC6 ultramicrotome) were stained with 2.5 % gadolinium(III) acetate (Sigma Aldrich) for 30 min, rinsed with ddH_2O and post-stained with 3% lead citrate (Leica Microsystems) for 8 minutes and rinsed again. Thin sections were analyzed using a Zeiss Libra 120 transmission electron microscope.

DNA extraction, PCR, and gene sequencing

Total DNA from amoeba cultures, symbionts and viruses were extracted using QIAGEN PowerSoil® DNA isolation kit or the QIAGEN Blood and Tissue kit®. The purified DNA served as a template for different PCR reactions, using the DreamTaq polymerase according to manufacturer's recommendations (Thermo Fisher Scientific). Primer sequences are given below.

To identify amoebae, 18S rRNA gene sequences were amplified with the primers 18SF and 18SR. To identify chlamydial symbionts, 16S rRNA gene sequences were amplified using the primers panCh16F2_mod and panCh16R2_mod. Custom-designed primer pair RNA_pol_viennavirus_81F and RNA_pol_viennavirus_3870R were used to amplify a 3789 bp fragment corresponding to the DNA-directed RNA polymerase subunit 2 of the *Viennavirus* isolate. The PCR product was used as a standard in the quantitative PCR assays.

Endpoint PCR mixtures were prepared in a final volume of 25 µL, containing 2.5 µL of 10X buffer, 200 nM of each deoxynucleotide triphosphate, 500 nM of each primer, 1.25 U of DreamTaq DNA polymerase, 2.5 µL of template DNA and molecular biology grade water in sufficient quantity for 25 µL. Amplifications were carried out with an initial denaturation at 95 °C for 2 min followed by 35 cycles of denaturation at 95 °C for 30 s, annealing at 65 °C for 30 s and extension at 72 °C for 1 min were carried out. The final extension was performed at 72 °C for 7 min.

The PCR reactions were checked, purified using QIAquick PCR purification kit (QIAGEN) and sequenced (Microsynth Austria). The resulting electropherograms were manually inspected using 4Peaks (5) and aligned with seaview (6). The BLASTn algorithm (7, 8) at the NCBI database nr was used for sequence similarity searches.

The primers pairs MCP_APolyphagaMimivirus_F/ MCP_APolyphagaMimivirus_R and MCP_Tupanvirusdo_F/ MCP_Tupanvirusdo_R were used to amplify the major capsid protein gene of *Tupanvirus deep ocean* and *Acanthamoeba* polyphaga m*imivirus* respectively, in digital droplet PCR (ddPCR) assays. The mastermix included 11 µl of 2 x QX200 ddPCR EvaGreen Supermix, 0.4 µl of each primer, 8.2 µl of molecular grade water and 2 µl of DNA sample. The cycling conditions were enzyme activation at 95 °C for 5 min, followed by 40 cycles of denaturation at 95 °C for 30 s and annealing 59 °C for 1 min, a final signal stabilization step at 4°C for 5 min, followed by 90°C for 5 min. A ramp rate of 2°C/s was used in all steps. The samples were analyzed with the QX200 Droplet reader

(Bio-Rad). Samples with less than 10.000 droplets were repeated. Finally, the software Quantasoft v.1.7.4.0917was used to calculate gene copy numbers.

Quantitative real-time PCR (qPCR) was performed by amplifying a 135 bp fragment of the DNA-directed RNA polymerase subunit 2 gene, using primer pair RNA_pol_viennavirus_2247F and RNA_pol_viennavirus_2382R. The master mix included 10 µl iQ™ SYBR® Green Supermix (Bio-Rad), 1 µl of each primer, and 7 µl of molecular grade water. The cycling conditions comprised an initial denaturation for 5 min at 95 °C, then 45 cycles of denaturation at 95 °C for 20 s, hybridization at 55 °C for 30 s, and elongation at 72 °C for 30 s. Gene copy numbers and qPCR efficiencies were calculated by referring to standard curves established for each experiment. All standard curves corresponding to the data that are shown in the results section had R^2 values higher than 0.99.

The ddPCR primers targeting the major capsid protein of *Acanthamoeba polyphaga mimivirus*, *Tupanvirus deep ocean* were designed using Primer3Plus (9). The primers were designed such that the primer length, Tm, and product size for each virus to be similar. Blastn (9, 10) searches adapted for short input sequences were performed to exclude that the primers target any of the *Acanthamoeba* hosts that were used in the experiments. This was confirmed by including negative controls during the ddPCR assay.

Viral replication was quantified with quantitative real-time PCR (qPCR) targeting the *Viennavirus* RNA polymerase gene, and with digital droplet PCR (ddPCR) targeting the respective major capsid protein gene of *Mimivirus* and *Tupanvirus*. Negative controls (amoeba without virus) were below the detection limit for all qPCR and ddPCR experiments

Primers and FISH probes used in this study

The following primers were used in this study: 18SF (5′-GTAGTCATATGCTTGTCTC-3′) and 18SR (5'-CGRARACCTTGTTACGAC-3'), annealing Temperatur 65 °C(11); RNApol_viennavirus_81F (5'-ACGAGACTTTACGAGGGCTT-3') and RNApol_viennavirus_3870R (5'-CGAGAGTTTTGTGGCGTAGG-3'), annealing Temperatur 65°C; RNApol_viennavirus_2247F (5'-GAAATCCTCTGTCGCCGTTC-3') and RNApol_viennavirus_2382R (5'-GCAAATGGGGCTCTTGTTCA-3'), annealing Temperatur 55°C; panCh16F2_mod (5'-CCGCCAACAYTGGGACT-3') and panCh16R2_mod (5'-GKAGGTRGCCGCYGCTTCTTTAC-3'), annealing Temperatur 65°C (12); MCP_ApolyphagaMimivirus_F (5'-TCGTTTTTACGAAACATGATGG-3') and MCP_ApolyphagaMimivirus_R (5'-CGATGGTGATTTGGAACACA-3'), annealing Temperatur 59°C; MCP_Tupanvirusdo_F (5'-AAAGGCCAATTGGTTCTCCT-3') and MCP_Tupanvirusdo_R (5'-ATCTCCGGAACGATTCACAG-3'), annealing Temperatur 59°C. The following FISH probes were used in this study: Euk516 (5'-GGAGGGCAAGTCTGGT-3') (13); Eub338-1 (5'-GCTGCCTCCCGTAGGAGT-3') (13), Eub338-2 (5'-GCACCCACCCGTAGGTGT-3') (4) and Eub338-3 (5'-GCTCCCACCCGTAGGTGT-3') (14);

Nag1088 (5'-GTGGCCCACGACAGCTTT-3') (14); Para581 (5'-ACTTATGTTCCCGCCTAC-3'); all at a hybridization Temperatur of 46°C.

Genome sequencing and annotation

Genomic DNA from purified *Viennavirus* particles and chlamydial symbionts obtained by passing through a 1.2 µm filter of amoeba culture supernatant, respectively, were obtained as described above. High molecular weight *Viennavirus* DNA was sequenced using the PacBio SMRT sequencing system by the DoE Joint Genome Institute. The *Viennavirus* raw reads were assembled using Flye v 2.4.2 (15), which produced a single contiguous and unambiguous sequence of 360961 nucleotides. The genome of the chlamydial symbiont was sequenced by generating paired-end (2 x 150 bp) reads using a Miseq platform. The quality of reads was analyzed using FastQC, and trimmed based on Q>30 using cutadapt v 1.9.1 (16). The assembly was performed using Spades v3.9.0 with standard settings (17).

Gene prediction and annotation for *Viennavirus* was done using Prokka v.1.14.6 (18). Additional predictions were done for non-coding RNAs using the Rfam database (19), and for proteins domains using the Pfam (20), SUPERFAMILY (21), and PROSITE (22) databases. Genome annotations were curated for genes of interest using the UniPro UGENE software (23).

For both genomes, reads were mapped back onto generated contigs in order to investigate coverage patterns, using Bowtie v 2.2.6 (24). This was further visualized using the Anv'io visualization tool and Blobtools in order to exclude any potential host-derived sequences based on coverage and sequence composition (25, 26). Refined genomes were then annotated in Anvi'o v 5.3 (27, 28).

For the visualization of the genome conservation between *P. acanthamoebae* UV-7 and *P. acanthamoebae* PAVD and *Marseillevirus marseillevirus* and *Viennavirus*, respectively, the genome sequences were blasted (v2.9.0) against each other. Only regions longer than 100 nt and a bitscore >100 were considered for visualization with circos-0.69 (29). Only the largest scaffold of *P. acanthamoebae* PAVD was included in the plot. Links represent regions with 100-99%, <99-90%, <90-80%, <80-70% identity, respectively. In addition the orientation of the genomic regions to the respective compared genome are represented by link colors.

Statistical analysis

Statistical analyses were carried out using R-studio (https://www.rstudio.com/, Version 4.1.3). The R-studio packages ggplot2 (30) and CAR (31) were used. When the prerequisites were fulfilled, ANOVA was used for multiple comparisons. Significant differences between the test groups (*A. castellanii*, *A. castellanii* + Viennavirus, *A. castellanii* + symbiont, *A. castellanii* + symbiont + Viennavirus) were found 12 hpi ($F_{3,8}$ = 7.15, $p < .01$) 24 hpi ($F_{3,8}$ =40.66, $p < .001$), 55 hpi ($F_{3,8}$ = 21.87, $p < .001$) and 90 hpi ($F_{3,8}$ = 22.48, $p < .001$). There was no significant difference detected at the beginning of the

experiment 0 hpi ($F_{3,8}$ = 0. 164 , p = 0.91). Student's t-test was used for pairwise comparisons.

Data availability

Sequences of the *Marseillvirus viennavirus* genome, the *Parachlamydia acanthamoebae* PAVD genome, and the 18S rRNA gene of *Acanthamoeba hatchetti* WWTP are available in the NCBI BioProject PRJNA799241. The *A. hatchetti* WWTP culture including the *Parachlamydia acanthamoebae* PAVD symbiont will availabe at the German Collection of Microorganisms and Cell Cultures DSMZ.

References

1. I. Lagkouvardos, J. Shen, M. Horn, Improved axenization method reveals complexity of symbiotic associations between bacteria and acanthamoebae. *Environ. Microbiol. Rep.* **6**, 383–388 (2014).

2. Y. Wang, R. M. Tian, Z. M. Gao, S. Bougouffa, P. Y. Qian, Optimal eukaryotic 18S and universal 16S/18S ribosomal RNA primers and their application in a study of symbiosis. *PLoS One* **9** (2014).

3. R. I. Amann, L. Krumholz, D. A. Stahl, Fluorescent-oligonucleotide probing of whole cells for determinative, phylogenetic, and environmental studies in microbiology. *J. Bacteriol.* **172**, 762–770 (1990).

4. H. Daims, A. Brühl, R. Amann, K. H. Schleifer, M. Wagner, The domain-specific probe EUB338 is insufficient for the detection of all Bacteria: development and evaluation of a more comprehensive probe set. *Syst. Appl. Microbiol.* **22**, 434–444 (1999).

5. A. Griekspoor, T. Groothuis, 4Peaks: a program that helps molecular biologists to visualize and edit their DNA sequence files v1. 7. (2005).

6. N. Galtier, M. Gouy, C. Gautier, SEAVIEW and PHYLO_WIN: two graphic tools for sequence alignment and molecular phylogeny. *Comput. Appl. Biosci.* **12**, 543–548 (1996).

7. N. C. Institute, National Cancer Institute, Basic Local Alignment Search Tool. *Definitions* (2020) https:/doi.org/10.32388/rhq6vj.

8. S. F. Altschul, W. Gish, W. Miller, E. W. Myers, D. J. Lipman, Basic local alignment search tool. *J. Mol. Biol.* **215**, 403–410 (1990).

9. A. Untergasser, *et al.*, Primer3Plus, an enhanced web interface to Primer3. *Nucleic Acids Research* **35**, W71–W74 (2007).

10. C. Camacho, *et al.*, BLAST+: architecture and applications. *BMC Bioinformatics* **10**, 421 (2009).

11. S. Schmitz-Esser, *et al.*, Diversity of bacterial endosymbionts of environmental acanthamoeba isolates. *Appl. Environ. Microbiol.* **74**, 5822–5831 (2008).

12. J. Lienard, *et al.*, Development of a New Chlamydiales-Specific Real-Time PCR and Its Application to Respiratory Clinical Samples. *J. Clin. Microbiol.* **49**, 2637–2642 (2011).

13. R. I. Amann, *et al.*, Combination of 16S rRNA-targeted oligonucleotide probes with flow cytometry for analyzing mixed microbial populations. *Appl. Environ. Microbiol.* **56**, 1919–1925 (1990).

14. D. Grimm, *et al.*, Development of 18S rRNA-targeted Oligonucleotide Probes for Specific Detection of Hartmannella and Naegleria in Legionella – positive Environmental Samples. *Syst. Appl. Microbiol.* **24**, 76–82 (2001).

15. M. Kolmogorov, J. Yuan, Y. Lin, P. A. Pevzner, Assembly of long, error-prone reads using repeat graphs. *Nat. Biotechnol.* **37**, 540–546 (2019).

16. M. Martin, Cutadapt removes adapter sequences from high-throughput sequencing reads. *EMBnet.journal* **17**, 10–12 (2011).

17. A. Bankevich, *et al.*, SPAdes: a new genome assembly algorithm and its applications to single-cell sequencing. *J. Comput. Biol.* **19**, 455–477 (2012).

18. T. Seemann, Prokka: rapid prokaryotic genome annotation. *Bioinformatics* **30**, 2068–2069 (2014).

19. I. Kalvari, *et al.*, Non-Coding RNA Analysis Using the Rfam Database. *Curr. Protoc. Bioinformatics* **62**, e51 (2018).

20. J. Mistry, *et al.*, Pfam: The protein families database in 2021. *Nucleic Acids Res.* **49**, D412–D419 (2021).

21. D. Wilson, *et al.*, SUPERFAMILY--sophisticated comparative genomics, data mining, visualization and phylogeny. *Nucleic Acids Res.* **37**, D380–6 (2009).

22. C. J. A. Sigrist, *et al.*, New and continuing developments at PROSITE. *Nucleic Acids Res.* **41**, D344–7 (2013).

23. K. Okonechnikov, O. Golosova, M. Fursov, UGENE team, Unipro UGENE: a unified bioinformatics toolkit. *Bioinformatics* **28**, 1166–1167 (2012).

24. B. Langmead, S. L. Salzberg, Fast gapped-read alignment with Bowtie 2. *Nat. Methods* **9**, 357–359 (2012).

25. A. M. Eren, *et al.*, Anvi'o: an advanced analysis and visualization platform for 'omics data. *PeerJ* **3**, e1319 (2015).

26. D. R. Laetsch, M. L. Blaxter, BlobTools: Interrogation of genome assemblies. *F1000Res.* **6**, 1287 (2017).

27. R. L. Tatusov, M. Y. Galperin, D. A. Natale, E. V. Koonin, The COG database: a tool for

genome-scale analysis of protein functions and evolution. *Nucleic Acids Res.* **28**, 33–36 (2000).

28. S. F. Altschul, W. Gish, W. Miller, E. W. Myers, D. J. Lipman, Basic local alignment search tool. *J. Mol. Biol.* **215**, 403–410 (1990).

29. M. Krzywinski, *et al.*, Circos: an information aesthetic for comparative genomics. *Genome Res.* **19**, 1639–1645 (2009).

30. C. Ginestet, ggplot2: Elegant Graphics for Data Analysis. *Journal of the Royal Statistical Society: Series A (Statistics in Society)* **174**, 245–246 (2011).

31. J. Fox, et al., Package “car”: Companion to applied regression. Wien: www. cran. r-project.org (2011).
